# Algorithmic academic framing in AI-driven smart libraries: a moderated mediation model of personalized learning and cognitive overload in academic decision-making

**DOI:** 10.3389/fpsyg.2026.1864163

**Published:** 2026-07-31

**Authors:** Yun Bai

**Affiliations:** Library, Jilin University of Finance and Economics, Changchun, China

**Keywords:** academic decision-making quality, AI self-efficacy, AI-driven smart libraries, algorithmic academic framing, cognitive overload, personalized learning

## Abstract

This study examines how AI-driven smart libraries are associated with academic decision-making by introducing the concept of algorithmic academic framing. Drawing on cognitive load theory and social cognitive theory, a conditional process model was developed in which algorithmic academic framing is associated with academic decision-making quality through personalized learning, while AI self-efficacy and cognitive overload serve as boundary conditions. Time-lagged data were collected from 328 students across three universities in China. Structural equation modeling results indicate that algorithmic academic framing is positively associated with personalized learning, which, in turn, is positively associated with academic decision-making quality. The indirect association is strengthened when AI self-efficacy is high and weakened when cognitive overload is elevated. These findings suggest that AI-driven academic systems may function as cognitive infrastructures whose impact appears to depend on both user capability and cognitive resource availability. The study advances theoretical understanding of algorithmic influence in higher education and provides practical guidance for the design and implementation of AI-driven library platforms.

## Introduction

The rapid integration of artificial intelligence into higher education has transformed how students search for, evaluate, and utilize academic information. AI-driven smart libraries now employ recommendation algorithms, adaptive search engines, and automated summarization tools to guide users through vast digital repositories. Prior research has highlighted the efficiency gains and personalization benefits associated with AI-driven educational technologies (Abinesh and Dulloo, 2024; Venkateswaran, 2023), suggesting that such systems enhance relevance, engagement, and learning outcomes. Yet, as digital infrastructures increasingly mediate academic cognition, an important question emerges: beyond improving access, how do algorithmic systems shape the very process of academic decision-making?

Recent scholarship on algorithmic influence argues that digital systems do not merely deliver information but frame attention, prioritize content, and subtly guide evaluative judgments (Vallabhaneni et al., 2024). In this regard, AI tools can be viewed as cognitive intermediaries that structure users’ perception of relevance and credibility. Although studies have examined recommendation systems in commercial and social media contexts (Strielkowski et al., 2025), comparatively limited attention has been devoted to their framing power within academic environments. Moreover, while personalization has been widely recognized as a key advantage of AI-supported learning systems (Kaswan et al., 2024), its downstream consequences for academic decision quality remain underexplored. This gap is particularly important because students’ academic judgments are not shaped only by the availability of information, but also by how information is encountered, judged as relevant, and cognitively processed during search and evaluation. Foundational work on information foraging and relevance suggests that users continuously assess informational cues, costs, and perceived value while navigating complex information environments (Pirolli and Card, 1999; Saracevic, 1996). Similarly, research on the information search process and search-as-learning emphasizes that searching is itself a learning and sensemaking activity rather than a neutral retrieval procedure (Kuhlthau, 1991; Marchionini, 2006). Thus, a critical gap persists in understanding the mechanism through which algorithmic systems influence consequential academic choices.

Importantly, existing research has primarily conceptualized AI-supported academic systems in terms of their technological functionalities, such as recommendation algorithms, adaptive search, algorithmic curation, or personalized content delivery (Kaswan et al., 2024; Peng and Li, 2025). Although these perspectives explain how AI systems generate and deliver information, they provide comparatively limited insight into how such outputs shape students’ interpretation of academic relevance, credibility, and priority during decision-making. In other words, recommendation systems describe what algorithms present (Kellogg et al., 2020), whereas considerably less attention has been devoted to understanding how algorithmically organized information cognitively frames students’ academic judgments.

This distinction is central to the present study. Algorithmic academic framing does not simply refer to personalization, curation, or decision support. Personalization concerns the perceived fit between system outputs and individual needs (Bernacki et al., 2021; Kaswan et al., 2024); curation concerns the selection, ranking, and organization of information within algorithmic environments (Kellogg et al., 2020); and decision support concerns assistance in evaluating alternatives and improving decision processes. By contrast, algorithmic academic framing refers to the cognitive interpretive influence through which AI-generated rankings, recommendations, summaries, and prompts make some sources, topics, and academic directions appear more relevant, credible, or worthy of attention than others (Hastuti, 2025). In this sense, the construct builds on, but is distinct from, choice architecture and nudging perspectives, which show how structured environments guide decisions by shaping salience and default attention (Thaler and Sunstein, 2008). Addressing this distinction requires moving beyond technology-centered explanations toward a cognitive framing perspective that captures how AI-mediated information environments influence academic sensemaking.

Building on these concerns, the present study introduces the concept of algorithmic academic framing to capture the extent to which AI-driven smart libraries shape students’ prioritization of sources, topics, and research directions. This perspective moves beyond usage-based explanations and aligns with broader calls to examine technology as a cognitive structuring force (Jose et al., 2025). It also connects with research on trust in automation and algorithmic reliance, which shows that users do not respond to algorithmic outputs uniformly; rather, they may accept, resist, or selectively rely on such outputs depending on perceived competence, transparency, and confidence in the system (Lee and See, 2004; Dietvorst et al., 2015; Logg et al., 2019). In addition, dual-process perspectives on judgment suggest that algorithmically prioritized cues may influence both rapid heuristic judgments and more deliberative academic evaluations (Kahneman, 2024). These perspectives help clarify why algorithmic academic framing is expected to influence personalized learning and academic decision-making quality, while also depending on user capability and cognitive resources. Importantly, research in cognitive load theory suggests that informational environments can either facilitate or hinder decision processes depending on the balance between guidance and overload (Gkintoni et al., 2025; Twabu, 2025). Therefore, while algorithmic framing may enhance personalized learning experiences, its effectiveness may be contingent upon users’ cognitive capacity and technological confidence.

Furthermore, social cognitive research emphasizes that individuals’ self-efficacy beliefs significantly shape how they interact with technological systems (Bergdahl and Sjöberg, 2025). Students who feel competent in navigating AI tools may better leverage algorithmic cues, whereas those experiencing cognitive overload may struggle to translate personalized recommendations into high-quality academic decisions. Integrating these perspectives responds to the research-oriented imperative to examine both enabling and constraining conditions of technology use (Wang et al., 2023). Accordingly, this study develops and tests a moderated mediation model in which algorithmic academic framing influences academic decision-making quality through personalized learning, with AI self-efficacy and cognitive overload serving as boundary conditions.

By empirically examining these relationships using time-lagged data from Chinese universities, the study addresses a theoretical and practical gap in the literature. It contributes to ongoing debates about the role of AI in higher education by demonstrating that the impact of smart libraries is not inherently beneficial or detrimental; rather, it depends on the interplay between algorithmic structuring, user capability, and cognitive demand. The study therefore positions algorithmic academic framing as a distinct cognitive mechanism through which AI-enabled smart libraries shape academic sensemaking, while differentiating it from adjacent constructs such as personalization, curation, nudging, and decision support. In doing so, the research advances a more nuanced understanding of AI-driven academic infrastructures and their implications for student decision processes (Alsbou and Alsaraireh, 2024; Mahamad et al., 2025).

## Literature review

### Algorithmic academic framing

The study posits that algorithmic academic framing captures the extent to which AI-driven smart library systems shape students’ academic sensemaking by prioritizing certain sources, topics, and evaluative cues, thereby influencing what appears relevant, credible, and worth pursuing. Rather than functioning as neutral repositories, contemporary smart libraries embed algorithmic ranking, recommendation, and summarization features that actively structure users’ information environments. Work on algorithmic influence and digital mediation suggests that such systems can guide attention, reduce search costs, and streamline evaluation by highlighting patterns and “best-fit” resources (Yeung, 2018). The notion is consistent with information foraging theory, which suggests that users navigate complex information environments by following cues that signal value, relevance, and expected payoff (Pirolli and Card, 1999). It also aligns with relevance theory in information science, where relevance is understood not as an objective property of information alone but as a judgment shaped by users’ goals, contexts, and interpretive needs (Saracevic, 1996). At the same time, scholars have cautioned that algorithmic outputs may narrow informational horizons or create dependence on pre-structured cues, especially when users treat recommendations as substitutes for critical appraisal (Kellogg et al., 2020; Gkintoni et al., 2025). Within academic contexts, this framing role becomes particularly consequential because students’ research decisions—such as selecting literature, defining research questions, or choosing methods—are strongly shaped by perceived relevance and credibility signals (Meng et al., 2025).

Although algorithmic academic framing shares certain characteristics with concepts such as algorithmic recommendation, algorithmic curation, and personalized content delivery, it represents a conceptually distinct perspective. Recommendation systems primarily describe the technological processes through which algorithms identify and present content based on user preferences or behavioral patterns (Kellogg et al., 2020; Peng and Li, 2025), whereas algorithmic curation focuses on the selection and organization of information within digital environments (Domínguez Figaredo, 2020; Hastuti, 2025). Likewise, personalized learning reflects learners’ perceptions that educational content is tailored to their individual needs (Kaswan et al., 2024). In contrast, algorithmic academic framing emphasizes the cognitive interpretive process through which AI-generated rankings, recommendations, and summaries shape students’ perceptions of what is academically relevant, credible, and worthy of further consideration. This distinction also connects with choice architecture and nudging perspectives, which show that the organization and presentation of options can influence attention, preference formation, and decision outcomes without directly removing choice (Thaler and Sunstein, 2008). Thus, the present construct extends beyond technological functionality by conceptualizing AI systems as cognitive framing mechanisms that influence academic sensemaking and subsequent decision processes.

From a cognitive perspective, algorithmic academic framing can be understood as a form of externally provided structure that helps learners manage complexity in information-rich environments. Research by Kaswan et al. (2024) on digital learning environments emphasizes that guidance tools can support effective learning by reducing unnecessary search effort and directing users toward task-relevant resources, thereby improving perceived fit between learner needs and available materials. Similarly, Kuhlthau’s information search process model suggests that users’ uncertainty, confidence, and sensemaking evolve during search, indicating that search is not merely a retrieval activity but a cognitively and emotionally situated learning process (Kuhlthau, 1991). Exploratory search research further argues that searching often involves moving from finding information toward understanding, especially in complex academic tasks (Marchionini, 2006). Extending this logic, algorithmic framing should enhance personalized learning because AI systems tailor resource suggestions based on user history, search patterns, and content similarity, which increases perceived relevance and facilitates efficient knowledge acquisition (Ayeni et al., 2024). However, the benefits of framing are unlikely to be uniform. Prior studies in algorithmic decision support propose that users’ engagement with algorithmic cues varies depending on how much they trust the system, how competent they feel using it, and whether they experience the system as supportive or overwhelming (Laak and Aru, 2025). This conditional view is supported by research on trust in automation, algorithm aversion, and algorithm appreciation, which indicates that users may rely on, resist, or selectively accept algorithmic recommendations depending on their perceptions of system reliability and their own confidence in using the system (Lee and See, 2004; Dietvorst et al., 2015; Logg et al., 2019).

In higher education, the theoretical significance of algorithmic academic framing lies in explaining how AI infrastructure influences academic outcomes through subtle, everyday interactions rather than through overt instruction. Existing studies have often operationalized AI library systems via adoption, frequency of use, or satisfaction metrics (Gulati and Unhelkar, 2024; Hu et al., 2024; Mahamad et al., 2025). Although valuable, such indicators provide limited insight into the cognitive pathway through which AI affects academic decisions. By focusing on framing, the present study aligns with calls to move beyond “technology as a tool” assumptions and to theorize AI as a socio-technical actor that shapes cognition and behavior (Adewojo et al., 2026). It also resonates with dual-process perspectives on judgment, which suggest that decision-making may be shaped both by fast cue-based evaluations and by slower deliberative reasoning (Kahneman, 2024). In AI-driven smart libraries, algorithmically prioritized cues may therefore influence students’ immediate perceptions of relevance while also shaping deeper academic evaluation. Accordingly, algorithmic academic framing is conceptualized as a cognitive framing mechanism embedded within system outputs—including rankings, prompts, recommendations, and summaries—that shapes students’ interpretation of academic relevance and, consequently, their academic judgments and preferences. This conceptualization provides a foundation for examining how algorithmic framing promotes personalized learning and, in turn, enhances the quality of academic decision-making, while acknowledging that the impact of such framing may depend on contextual and individual boundary conditions.

### Linking algorithmic academic framing with personalized learning

Algorithmic academic framing is theorized to enhance personalized learning by structuring how students interpret and prioritize academic information within AI-driven smart libraries. Domínguez Figaredo (2020) argued that algorithmic systems operate as regulatory architectures that subtly guide user behavior by shaping exposure and salience. Similarly, Upadhyay (2025) documented how algorithmic outputs influence attention allocation and evaluative judgment in organizational contexts, suggesting that algorithms function not merely as tools but as framing devices. Extending this logic to educational environments, Omoyemi and Adefisayo (2025) demonstrated that perceived personalization in digital systems significantly increases learning engagement and perceived relevance. In addition, adaptive learning research has consistently shown that when technological systems align content recommendations with individual needs, learners experience greater cognitive fit and instructional effectiveness.

Further empirical evidence supports the notion that recommendation algorithms enhance perceived informational relevance (Laak and Aru, 2025; Zeng et al., 2022). For example, prior work on digital recommendation systems found that algorithmic curation increases users’ confidence in content selection and improves task efficiency. Moreover, studies in educational technology contexts reveal that tailored feedback and adaptive resource suggestions strengthen learners’ perception of individualized support, thereby improving learning outcomes (Peng and Li, 2025; Strielkowski et al., 2025). From a cognitive standpoint, structured guidance reduces search ambiguity and directs attention toward task-relevant materials, which fosters deeper engagement with academic content (Twabu, 2025). Collectively, these findings suggest that algorithmic academic framing, by organizing and prioritizing information flows, should positively influence personalized learning experiences.

Accordingly, the following hypothesis is proposed:

*H1*: Algorithmic academic framing is positively related to personalized learning.

### Linking personalized learning with academic decision-making quality

Personalized learning is expected to play a central role in shaping the quality of students’ academic decisions. Research on adaptive educational systems has long suggested that when learners perceive instructional content as tailored to their needs, their cognitive engagement and task performance improve. Li (2023) that perceived personalization enhances learning satisfaction and effectiveness in digital environments. In this thread, subsequent investigations confirmed that adaptive recommendation systems improve users’ perceived relevance of materials and task alignment (Schmid et al., 2022; Taylor et al., 2025). Importantly, relevance perception is a critical antecedent of decision quality because individuals are more likely to invest cognitive effort in evaluating information that appears directly applicable to their goals.

Decision-making research further indicates that structured and contextually aligned information reduces uncertainty and facilitates more accurate judgments. For instance, Bernacki et al. (2021) found that access to organized and meaningful information improves evaluative confidence and decision effectiveness, particularly in knowledge-intensive tasks. In educational settings, students who perceive stronger alignment between available resources and their academic objectives tend to exhibit more deliberate information processing and higher-quality academic choices (Dumont and Ready, 2023; Silva et al., 2024). Moreover, research grounded in information processing theory suggests that tailored informational inputs enhance cognitive coherence, enabling individuals to integrate evidence more systematically when making decisions (Tam and Ho, 2006).

Additionally, studies examining digital learning environments have shown that personalization reduces extraneous search costs and supports goal-oriented reasoning, thereby improving strategic academic planning (El Mhouti et al., 2013; Nguyen et al., 2024). When learners receive recommendations that reflect their interests and prior activity, they are better positioned to evaluate alternatives and select appropriate research directions (Li, 2023). Taken together, these findings indicate that personalized learning serves as a cognitive mechanism through which AI-driven systems enhance academic judgment.

Accordingly, the following hypothesis is proposed:

*H2*: Personalized learning is positively related to academic decision-making quality.

Subsequently, algorithmic academic framing is likely to enhance academic decision-making quality indirectly through personalized learning. The study anticipates that as AI-driven smart libraries prioritize and recommend resources based on users’ profiles and search behaviors, they structure informational environments in ways that increase perceived relevance and alignment with academic goals. Such framing mechanisms can reduce ambiguity and streamline cognitive processing, thereby fostering stronger personalized learning experiences. In turn, research on adaptive and technology-enhanced learning consistently shows that perceived personalization improves evaluative confidence, engagement, and decision effectiveness in educational contexts (Bernacki et al., 2021; Zhang et al., 2020). For instance, prior studies have demonstrated that personalization functions as a key mechanism linking digital system features to performance outcomes (Ingkavara et al., 2022; Shamsudin and Cao, 2025; Zheng et al., 2022). On the other hand, Ausel et al. (2025); Kezar et al. (2023) has confirmed that tailored instructional support enhances higher-order academic judgments. Drawing on this reasoning, algorithmic academic framing is expected to influence academic decision-making quality primarily through its ability to generate personalized learning experiences. Accordingly, personalized learning is proposed to mediate the relationship between algorithmic academic framing and academic decision-making quality. Hence,

*H3*: Personalized learning mediates the relationship between Algorithmic academic framing and academic decision-making quality.

### Moderating role of AI self-efficacy

Although algorithmic academic framing may enhance personalized learning, its effectiveness is unlikely to be uniform across individuals. Social cognitive theory emphasizes that self-efficacy shapes how individuals interpret, engage with, and benefit from technological systems (Bandura, 2001). Bandura (2014) argued that efficacy beliefs influence cognitive effort, persistence, and the extent to which individuals translate external cues into meaningful action. In technology-mediated contexts, Khan et al. (2024) demonstrated that higher computer self-efficacy predicts deeper engagement and more effective use of digital tools. Similarly, subsequent research found that users with stronger technological self-confidence are more capable of leveraging system features to enhance task performance (He, 2025). In educational settings, studies have shown that students with higher digital or AI-related self-efficacy exhibit greater adaptability and stronger learning outcomes when interacting with intelligent tutoring systems (Chou et al., 2025). Moreover, research on AI-supported environments suggests that perceived competence strengthens users’ trust in algorithmic outputs and increases their willingness to rely on system recommendations (Hui et al., 2025). From a cognitive standpoint, efficacy beliefs enable individuals to interpret algorithmic cues as supportive guidance rather than opaque directives (Wu et al., 2025), thereby intensifying the framing effect of AI systems. This reasoning is also consistent with broader digital-behavior research showing that control-related beliefs can condition how psychological states are translated into technology-use patterns (Chu et al., 2025).

Building on this reasoning, AI self-efficacy is expected to strengthen the positive relationship between algorithmic academic framing and personalized learning. When students feel confident in their ability to use AI-driven smart libraries, they are more likely to meaningfully engage with algorithmic recommendations and convert them into tailored learning experiences. Conversely, lower AI self-efficacy may weaken this translation process. Therefore:

*H4*: AI self-efficacy positively moderates the relationship between algorithmic academic framing and personalized learning, such that the relationship is stronger at higher levels of AI self-efficacy.

Extending this logic, if AI self-efficacy strengthens the first-stage relationship, it should also condition the indirect effect of algorithmic academic framing on academic decision-making quality via personalized learning. Moderated mediation research suggests that when a moderator influences the initial pathway of a mediation model, the overall indirect effect becomes contingent upon that moderator (Wang and Preacher, 2015). Empirical evidence across digital learning contexts supports this pattern, demonstrating that the performance benefits of technology features are amplified when users possess high self-efficacy (Chou et al., 2025; He, 2025; Hui et al., 2025). Accordingly:

*H5*: The indirect effect of algorithmic academic framing on academic decision-making quality through personalized learning is stronger when AI self-efficacy is high.

### Moderating role of cognitive overload

While personalization may enhance academic decision-making quality, its benefits can be undermined when students experience excessive cognitive demands. Cognitive load theory posits that individuals possess limited processing capacity, and when informational demands exceed this capacity, learning and judgment quality deteriorate (Gkintoni et al., 2025; Huang and Xu, 2025). Sweller’s foundational work emphasized that extraneous load interferes with meaningful processing, thereby impairing higher-order reasoning (2011). Subsequent research in multimedia and digital learning environments confirmed that information saturation and interface complexity reduce comprehension and evaluative accuracy (Mehta et al., 2025). For instance, studies have shown that excessive recommendations or densely structured dashboards can overwhelm users, leading to superficial processing and poorer decision outcomes (Meng et al., 2025; Twabu, 2025; Wu et al., 2025). Moreover, evidence from technology-mediated contexts indicates that cognitive overload diminishes the effectiveness of adaptive systems, as users struggle to translate personalized inputs into coherent judgments (Lin et al., 2024). Research on decision-making similarly demonstrates that cognitive strain reduces deliberative evaluation and increases reliance on heuristics (Khan et al., 2024), which may weaken the quality of academic choices. In educational technology settings, scholars have further observed that personalization benefits are contingent upon manageable informational load, suggesting that cognitive overload operates as a boundary condition (Lin et al., 2024; Mehta et al., 2025).

Drawing on this body of work, cognitive overload is expected to weaken the positive association between personalized learning and academic decision-making quality. When students experience lower levels of cognitive strain, they are better able to integrate personalized recommendations into systematic evaluation and informed academic decisions. However, under high cognitive overload, the advantages of personalization may be attenuated due to constrained processing capacity. Therefore:

*H6*: Cognitive overload negatively moderates the relationship between personalized learning and academic decision-making quality, such that the relationship is weaker at higher levels of cognitive overload.

Furthermore, because cognitive overload influences the second stage of the mediation process, the overall indirect effect of algorithmic academic framing on academic decision-making quality via personalized learning should vary across levels of cognitive overload. Moderated mediation logic suggests that when a downstream path is contingent upon a moderator, the strength of the indirect effect becomes conditional (Wang and Preacher, 2015). Accordingly:

*H7*: The indirect effect of algorithmic academic framing on academic decision-making quality through personalized learning is weaker when cognitive overload is high.

Taken together, cognitive load theory and social cognitive theory provide complementary explanations for the proposed model. Cognitive load theory posits that learning outcomes depend on learners’ ability to efficiently process instructional information while minimizing unnecessary cognitive demands (Sweller, 1988, 2011), a concern echoed in recent online-learning research linking excessive learning demands with student burnout (Tu et al., 2026). Within AI-driven smart library environments, personalized learning represents the primary mechanism through which algorithmically structured information may facilitate learners’ processing of relevant academic resources by reducing unnecessary search effort and increasing perceived relevance (Sun et al., 2008; Kaswan et al., 2024). In contrast, social cognitive theory emphasizes that individuals differ in their capability to benefit from technological environments according to their self-efficacy beliefs (Bandura, 1986; Compeau and Higgins, 1995). At the same time, cognitive load theory suggests that excessive cognitive demands may diminish the benefits of personalized learning by limiting learners’ capacity to effectively process and apply algorithmically recommended information (Sweller, 2011; Zha et al., 2023). Consequently, personalized learning serves as the primary explanatory mechanism linking algorithmic academic framing with academic decision-making, whereas AI self-efficacy and cognitive overload represent theoretically complementary enabling and constraining boundary conditions.

**Figure 1:**
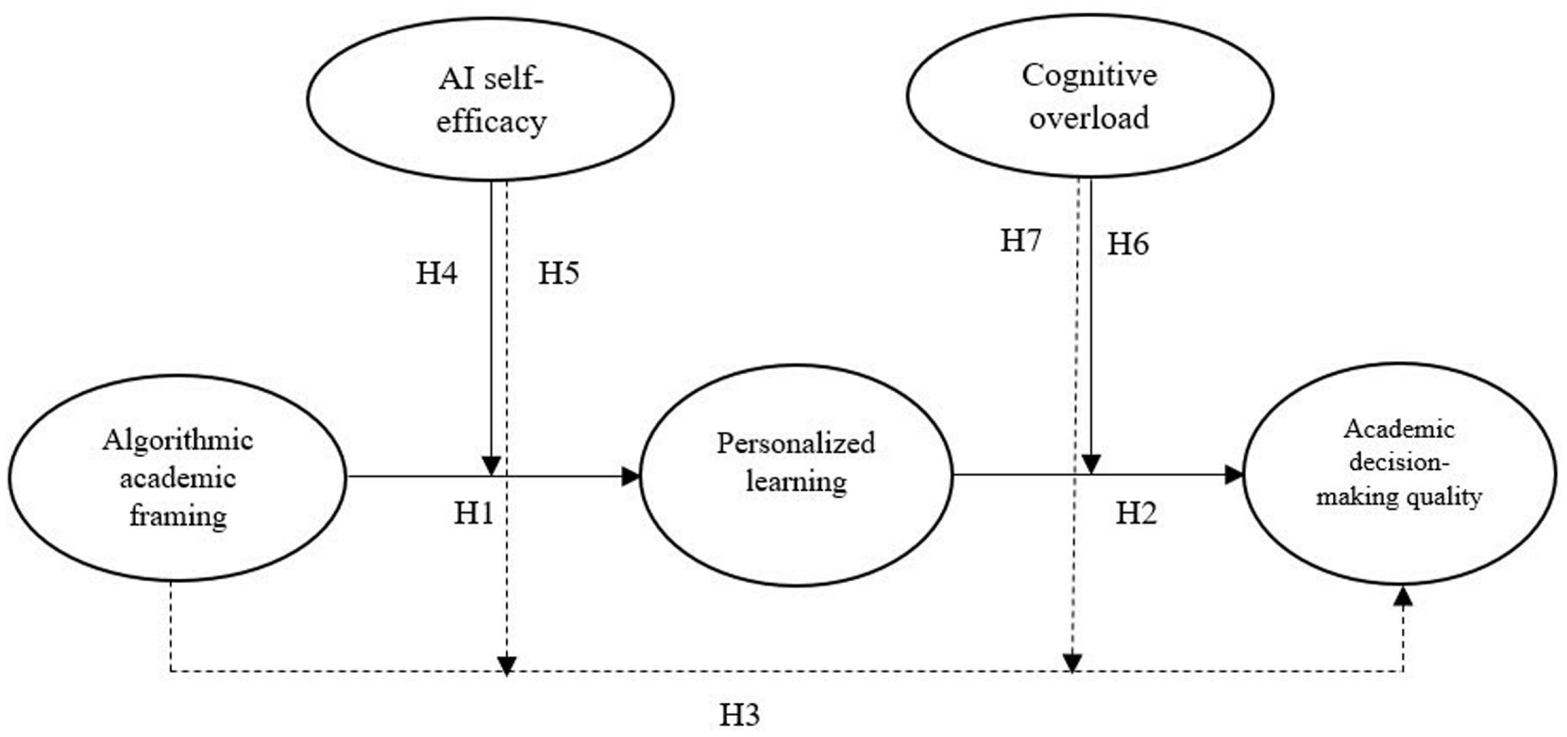
Conceptual model.

Figure 1 shows the conceptual model.

## Method

Data were collected from undergraduate and postgraduate students enrolled in three comprehensive universities in eastern and central China, where AI-driven smart library systems (e.g., intelligent search engines, recommendation algorithms, and automated citation tools) are routinely integrated into academic activities. A time-lagged survey design was employed to reduce common method bias. The two-wave design was also intended to establish temporal separation between predictor/boundary-condition variables and subsequent learning and decision-related perceptions. At Time 1, algorithmic academic framing, AI self-efficacy, and demographic variables were measured. At Time 2, four weeks later, personalized learning, cognitive overload, and academic decision-making quality were measured. The four-week interval was selected because it was long enough to reduce immediate consistency bias and short-term recall effects, while remaining short enough to ensure that students’ use of AI-driven smart library systems and academic tasks remained relatively stable across waves. After obtaining institutional approval from department coordinators and library administrators, the researchers physically approached students in library reading halls, digital resource centers, and postgraduate research rooms. Participants were informed about the purpose of the study, assured of anonymity and voluntary participation, and provided with a brief cover letter explaining that the research aimed to understand how AI-supported library systems influence academic learning and decision-making processes. No personally identifiable information was collected, and respondents were told they could withdraw at any time. No financial compensation was provided for participation.

A purposive sampling strategy was adopted to ensure that participants had prior experience using AI-driven smart library features, as this was essential for evaluating algorithmic academic framing and personalized learning experiences. Participants with prior experience were intentionally selected because meaningful evaluation of algorithmic academic framing required direct interaction with AI-driven smart library functionalities. Consequently, the findings should be interpreted within the context of users who possess at least a basic level of familiarity with such systems. Questionnaires were distributed in paper form during scheduled study periods, and respondents completed them on-site to maximize response quality and reduce non-response bias. At Time 1, 420 questionnaires were distributed and 356 were returned. Thus, 64 students either declined participation or did not return usable Time 1 questionnaires, yielding an initial response rate of 84.76%. Time 1 measured algorithmic academic framing, AI self-efficacy, and demographic variables. Four weeks later, the same participants were re-contacted in person and invited to complete the second survey. To match responses across waves while preserving anonymity, participants generated a unique self-created identification code and entered the same code on both questionnaires. This procedure allowed the researchers to link Time 1 and Time 2 responses without collecting names, student numbers, or other personally identifiable information. At Time 2, 341 questionnaires were collected, of which 328 matched correctly with Time 1 responses and were usable after screening for missing data and response patterns. The final matched sample therefore represented 92.13% of Time 1 respondents and 78.10% of the initially distributed questionnaires. The overall attrition/exclusion from Time 1 returned questionnaires to the final matched sample was 7.87%. Thirteen Time 2 questionnaires were excluded because they could not be matched accurately or showed incomplete/invalid response patterns.

To assess potential attrition bias, we compared retained participants in the final matched sample (*n* = 328) with participants who completed Time 1 but were not retained in the final matched dataset (*n* = 28). Independent-samples t-tests indicated no significant differences between retained and lost participants in algorithmic academic framing or AI self-efficacy, and chi-square tests showed no significant differences in gender, academic level, discipline, or AI smart library usage frequency. These results suggest that attrition was unlikely to systematically bias the final sample. In addition, because questionnaires were completed on-site and participants were recruited from multiple academic locations within each university, nonresponse bias was minimized.

The demographic profile indicated that 52% of respondents were female and 48% were male. Approximately 61% were undergraduates and 39% were postgraduate students. In terms of academic discipline, 34% were from social sciences, 29% from engineering and technology, 21% from natural sciences, and 16% from humanities and arts. Regarding frequency of AI smart library usage, 27% reported daily use, 46% weekly use, and 27% occasional use. The average age of participants was 22.8 years (SD = 2.4). This diverse and sufficiently large sample enhances the generalizability and robustness of the empirical findings within the Chinese higher education context.

## Measures

All constructs were measured using previously validated scales adapted to the context of AI-driven smart libraries in higher education. Following Brislin (1980) translation/back-translation procedure, the original English items were translated into Chinese by two bilingual researchers and then back-translated into English by an independent bilingual expert. Minor wording differences were discussed and resolved through consensus to ensure semantic equivalence, cultural appropriateness, and clarity for Chinese university students.

Because no established scale was available to directly measure algorithmic academic framing in AI-driven smart library contexts, the six items were contextually adapted from the conceptual literature on algorithmic influence and framing in digital environments (Kellogg et al., 2020; Yeung, 2018). Specifically, the adaptation involved rewording conceptually relevant statements to reflect how AI-driven smart library systems shape students’ perceptions of academic relevance, credibility, and prioritization while preserving consistency with the theoretical definition of the construct. The item development process involved three steps. First, the authors reviewed conceptual work on algorithmic influence, digital framing, recommendation systems, and AI-mediated information environments to identify the core dimensions of algorithmic academic framing. Second, an initial item pool was reviewed by three experts in educational technology, library and information science, and higher education research to assess content relevance, clarity, and contextual fit. Third, the revised items were cognitively pretested with a small group of students who had prior experience using AI-driven smart library systems, and minor wording refinements were made before the main survey.

Algorithmic academic framing was measured using six items adapted from research on algorithmic influence and framing in digital environments (Kellogg et al., 2020; Yeung, 2018). Items captured the extent to which AI-based library systems shape students’ perceptions of relevance and importance. A sample item is: “The smart library’s AI recommendations influence how I prioritize academic sources.” Based on the expert panel ratings, item-level content validity indices for the AAF items ranged from 0.83 to 1.00, and the scale-level content validity index was 0.94, indicating strong content validity. To minimize content overlap with personalized learning and academic decision-making quality, the algorithmic academic framing items focused specifically on how AI-generated rankings, recommendations, prompts, and summaries shape students’ perceptions of relevance, credibility, and priority. In contrast, personalized learning items focused on perceived tailoring of learning resources to individual needs, while academic decision-making quality items focused on the perceived quality of students’ final academic judgments and choices. Thus, the measures were designed to capture distinct stages of the proposed process: algorithmic framing as the upstream interpretive cue, personalized learning as the perceived learning experience, and decision-making quality as the downstream evaluative outcome.

Personalized learning was assessed with five items adapted from technology-enhanced personalized learning research (Sun et al., 2008), reflecting the perceived tailoring of content to individual needs. A sample item is: “The AI library system provides learning resources that match my academic interests”. Academic decision-making quality was measured with five items adapted from decision effectiveness scales in educational contexts (Scott and Bruce, 1995), with a sample item: “I make well-informed academic decisions when selecting research materials”. AI self-efficacy was measured using four items adapted from computer self-efficacy scales (Compeau and Higgins, 1995), for example, “I feel confident in my ability to use AI-based library tools effectively.”

Cognitive overload was assessed with four items adapted from cognitive load measures (Sweller, 2011), such as: “Using the AI library system sometimes overwhelms me with too much information.” In line with the study context, cognitive overload was operationalized as students’ perceived overall overload when interacting with AI-driven smart library systems, particularly overload arising from excessive information, recommendation density, and interface complexity. Therefore, the measure captured general perceived cognitive strain rather than separately modeling intrinsic, extraneous, and germane cognitive load components. This operationalization is consistent with prior cognitive load and mental workload research that treats perceived overload as a subjective indicator of limited processing capacity in complex learning and decision environments (Leppink et al., 2013; Hart and Staveland, 1988). The complete set of measurement items for all constructs is provided in Appendix A. All items were rated on a five-point Likert scale ranging from 1 = strongly disagree to 5 = stronlgy agree.

## Results

The data were screened and analyzed using IBM SPSS AMOS with a covariance-based structural equation modeling approach. The measurement and structural models were estimated using maximum likelihood estimation. Prior to model estimation, the dataset was examined for missing values, response patterns, and distributional assumptions. Cases with substantial missing responses, unmatched Time 1–Time 2 identification codes, or patterned responses were removed during the screening process; therefore, the final matched dataset consisted of 328 usable responses. Missingness was limited and primarily related to unmatched Time 1–Time 2 codes and incomplete Time 2 responses rather than systematic item nonresponse. Little’s MCAR test was non-significant, χ^2^ = 41.27, df = 38, *p* = 0.326, suggesting that the missingness pattern did not significantly deviate from missing completely at random. To examine whether the results were sensitive to listwise deletion, the structural model was re-estimated using FIML-based estimation. The direction, magnitude, and statistical significance of the hypothesized paths remained substantively unchanged, indicating that missing-data handling did not materially affect the findings. Skewness and kurtosis values were inspected and fell within commonly accepted limits for SEM, suggesting that severe non-normality was not present. Nevertheless, because indirect and conditional indirect effects may be sensitive to distributional assumptions, bias-corrected bootstrapping with 5,000 resamples was used to estimate confidence intervals for mediation and moderated mediation effects.

Latent interaction effects were estimated using the product-indicator approach in AMOS. Before creating the interaction terms, the indicators of algorithmic academic framing and AI self-efficacy were mean-centered, and product indicators were then created to represent the algorithmic academic framing × AI self-efficacy interaction. The same procedure was applied to the personalized learning and cognitive overload indicators to construct the PL × cognitive overload interaction. This approach is appropriate for testing latent interactions in covariance-based SEM because it allows the interaction to be modeled while accounting for measurement error in the underlying constructs (Marsh et al., 2004; Little et al., 2006). The interaction models were evaluated by examining the significance of the latent interaction paths, ΔR^2^ attributable to each interaction step, bootstrapped confidence intervals, and the stability of the substantive paths relative to the baseline structural model. Because the product-indicator approach in AMOS retains conventional SEM estimation, global fit indices were inspected for the interaction model; however, interpretation primarily emphasized interaction coefficients, ΔR^2^, f^2^, bootstrapped confidence intervals, and path stability. The model including interaction terms retained acceptable overall fit and produced theoretically consistent estimates, indicating that the moderation effects could be interpreted meaningfully.

Effect sizes were computed using Cohen’s f^2^ in the latent-variable context by comparing the explained variance of the relevant endogenous construct in models with and without the focal predictor. Specifically, f^2^ was calculated as: f^2^ = (R^2^included − R^2^excluded) / (1 − R^2^included). Following Cohen (1988) guidelines, f^2^ values of approximately 0.02, 0.15, and 0.35 were interpreted as small, medium, and large effects, respectively. This procedure allowed the substantive contribution of each direct and interaction effect to be evaluated beyond statistical significance.

Table 1 reports the descriptive statistics, internal consistency reliabilities, and correlations among the study variables. All constructs demonstrated satisfactory reliability, with Cronbach’s alpha values ranging from 0.873 to 0.912, exceeding the 0.700 threshold. In addition to Cronbach’s alpha, McDonald’s omega was calculated to provide a more robust estimate of internal consistency. Omega values were also satisfactory: algorithmic academic framing = 0.931, personalized learning = 0.940, academic decision-making quality = 0.946, AI self-efficacy = 0.904, and cognitive overload = 0.891. These values further confirmed the reliability of the constructs. Algorithmic academic framing was positively correlated with personalized learning (r = 0.612, *p* < 0.001) and academic decision-making quality (r = 0.548, *p* < 0.001). Personalized learning also showed a strong positive association with academic decision-making quality (r = 0.639, *p* < 0.001). AI self-efficacy was positively related to algorithmic academic framing (r = 0.476, *p* < 0.001) and personalized learning (r = 0.521, *p* < 0.001). In contrast, cognitive overload exhibited significant negative correlations with personalized learning (r = −0.342, *p* < 0.001) and academic decision-making quality (r = −0.371, *p* < 0.001), supporting its theorized constraining role. None of the correlations exceeded 0.700, suggesting that multicollinearity is unlikely to be a concern in subsequent structural analyses.

**Table 1:** Descriptive statistics, reliability, and correlations.

Variable	Mean	SD	1	2	3	4	5
1. Algorithmic academic framing (AAF)	3.742	0.681	0.901				
2. Personalized learning (PL)	3.865	0.654	0.612***	0.894			
3. Academic decision-making quality (ADQ)	3.918	0.623	0.548***	0.639***	0.912		
4. AI self-efficacy (AISE)	3.776	0.702	0.476***	0.521***	0.498***	0.887	
5. Cognitive overload (CO)	3.214	0.744	−0.219**	−0.342***	−0.371***	−0.268***	0.873

*N* = 328. Diagonal values represent Cronbach’s alpha. **p < 0.01. ***p < 0.001.

Table 2 presents the confirmatory factor analysis results. All standardized factor loadings ranged from 0.798 to 0.902, exceeding the recommended threshold of 0.700, indicating strong indicator reliability. Composite reliability values ranged from 0.889 to 0.931, surpassing the suggested minimum of 0.700. The average variance extracted values ranged from 0.668 to 0.771, exceeding the 0.500 criterion, demonstrating adequate convergent validity. Together with the McDonald’s omega values reported above, these results indicate that reliability was not dependent only on Cronbach’s alpha and that the constructs demonstrated strong internal consistency across multiple reliability criteria. These findings indicate that the measurement model exhibits satisfactory internal consistency and convergent validity, supporting the suitability of the constructs for subsequent structural model testing.

**Table 2:** Measurement model results.

Construct	Item	Standardized loading	CR	AVE
Algorithmic academic framing (AAF)	AAF1	0.821	0.918	0.692
	AAF2	0.856		
	AAF3	0.844		
	AAF4	0.801		
	AAF5	0.838		
	AAF6	0.829		
Personalized learning (PL)	PL1	0.873	0.926	0.758
	PL2	0.891		
	PL3	0.862		
	PL4	0.854		
	PL5	0.869		
Academic decision-making quality (ADQ)	ADQ1	0.884	0.931	0.771
	ADQ2	0.902		
	ADQ3	0.873		
	ADQ4	0.861		
	ADQ5	0.889		
AI self-efficacy (AISE)	AISE1	0.842	0.903	0.701
	AISE2	0.865		
	AISE3	0.816		
	AISE4	0.829		
Cognitive overload (CO)	CO1	0.801	0.889	0.668
	CO2	0.844		
	CO3	0.832		
	CO4	0.798		

All factor loadings are significant at *p* < 0.001.

Table 3 reports the discriminant validity assessment using the Fornell–Larcker criterion. The square root of the average variance extracted for each construct (shown on the diagonal) exceeds its corresponding inter-construct correlations. For instance, the square root of AVE for algorithmic academic framing (0.832) is greater than its correlations with personalized learning (0.612), academic decision-making quality (0.548), AI self-efficacy (0.476), and cognitive overload (−0.219). Similar patterns are observed for all other constructs. To complement the Fornell–Larcker criterion, HTMT ratios were also examined. All HTMT values were below the conservative 0.850 threshold, supporting discriminant validity (Table 4). These results indicate that the constructs were empirically distinguishable despite their conceptual proximity.

**Table 3:** Discriminant validity (Fornell–Larcker criterion).

Construct	AAF	PL	ADQ	AISE	CO
Algorithmic academic framing (AAF)	**0.832**				
Personalized learning (PL)	0.612	**0.870**			
Academic decision-making quality (ADQ)	0.548	0.639	**0.878**		
AI self-efficacy (AISE)	0.476	0.521	0.498	**0.837**	
Cognitive overload (CO)	−0.219	−0.342	−0.371	−0.268	**0.817**

Diagonal elements (bold) represent the square root of the average variance extracted (AVE).

**Table 4:** Discriminant validity (HTMT ratio).

Construct pair	HTMT
AAF–PL	0.682
AAF–ADQ	0.605
AAF–AISE	0.532
AAF–CO	0.247
PL–ADQ	0.708
PL–AISE	0.585
PL–CO	0.387
ADQ–AISE	0.554
ADQ–CO	0.416
AISE–CO	0.305

AAF = algorithmic academic framing; PL = personalized learning; ADQ = academic decision-making quality; AISE = AI self-efficacy; CO = cognitive overload.

Discriminant validity was also evaluated beyond statistical thresholds. Algorithmic academic framing items captured students’ perceptions of how AI-generated rankings, recommendations, summaries, and prompts shape judgments of relevance, credibility, and priority. Personalized learning items captured the perceived fit between AI-supported resources and students’ individual learning needs, whereas academic decision-making quality items captured the perceived quality of students’ final academic judgments and choices. Thus, the three conceptually related constructs represent different stages of the proposed process: algorithmic academic framing as the upstream interpretive cue, personalized learning as the intermediate learning experience, and academic decision-making quality as the downstream evaluative outcome. These results indicate that each construct shares more variance with its own indicators than with other constructs, supporting adequate discriminant validity of the measurement model.

Cross-loading diagnostics were further examined to assess item-level distinctiveness. All items loaded most strongly on their intended latent construct, and no item showed a higher loading on a non-target construct. This evidence is particularly important for distinguishing algorithmic academic framing from personalized learning and academic decision-making quality (Table 5).

**Table 5:** Discriminant validity (cross-loadings).

Item	AAF	PL	ADQ	AISE	CO
AAF1	**0.821**	0.458	0.411	0.372	−0.181
AAF2	**0.856**	0.491	0.439	0.405	−0.201
AAF3	**0.844**	0.463	0.421	0.389	−0.186
AAF4	**0.801**	0.438	0.397	0.361	−0.174
AAF5	**0.838**	0.471	0.426	0.394	−0.193
AAF6	**0.829**	0.466	0.418	0.386	−0.188
PL1	0.501	**0.873**	0.536	0.451	−0.286
PL2	0.521	**0.891**	0.558	0.474	−0.303
PL3	0.488	**0.862**	0.522	0.442	−0.274
PL4	0.496	**0.854**	0.531	0.456	−0.291
PL5	0.503	**0.869**	0.541	0.462	−0.295
ADQ1	0.432	0.557	**0.884**	0.426	−0.318
ADQ2	0.461	0.579	**0.902**	0.455	−0.337
ADQ3	0.421	0.541	**0.873**	0.419	−0.304
ADQ4	0.414	0.529	**0.861**	0.407	−0.291
ADQ5	0.449	0.562	**0.889**	0.438	−0.326
AISE1	0.398	0.442	0.415	**0.842**	−0.241
AISE2	0.421	0.478	0.446	**0.865**	−0.268
AISE3	0.384	0.436	0.402	**0.816**	−0.226
AISE4	0.407	0.459	0.428	**0.829**	−0.252
CO1	−0.182	−0.287	−0.315	−0.241	**0.801**
CO2	−0.206	−0.318	−0.346	−0.263	**0.844**
CO3	−0.194	−0.302	−0.331	−0.249	**0.832**
CO4	−0.221	−0.327	−0.358	−0.271	**0.798**

AAF = algorithmic academic framing; PL = personalized learning; ADQ = academic decision-making quality; AISE = AI self-efficacy; CO = cognitive overload. Boldface values represent the primary factor loadings of each indicator on its corresponding construct.

To assess discriminant validity and compare alternative specifications, several competing measurement models were tested using confirmatory factor analysis (Table 6). The one-factor model, in which all items loaded onto a single latent construct, demonstrated poor fit (CFI = 0.621, TLI = 0.587, RMSEA = 0.098, SRMR = 0.093), indicating substantial model misfit. Fit improved progressively as factors were separated. The hypothesized five-factor model exhibited the best fit to the data (χ^2^/df = 1.550, CFI = 0.962, TLI = 0.956, RMSEA = 0.041, SRMR = 0.036), outperforming all alternative models. The substantial improvement in fit indices supports the distinctiveness of algorithmic academic framing, personalized learning, academic decision-making quality, AI self-efficacy, and cognitive overload. To further assess the potential influence of common method bias, a common latent factor (CLF) analysis was conducted. The inclusion of the common latent factor resulted in only negligible changes in model fit (ΔCFI = 0.002, ΔRMSEA = 0.001, ΔSRMR = 0.001), suggesting that common method variance was unlikely to substantially influence the estimated relationships. Recognizing that the CLF test alone is not a definitive diagnostic, an additional unmeasured latent method factor model with constrained method loadings was estimated as a robustness check. The substantive path coefficients remained stable in direction and significance after including the constrained method factor, and no hypothesized relationship changed materially. Together with the two-wave design and the temporal separation of predictor and outcome variables, these results suggest that common method bias was unlikely to account for the observed relationships. These findings provide further evidence of adequate discriminant validity.

**Table 6:** Comparison of alternative measurement models.

Model	Factor structure specification	χ^2^	df	χ^2^/df	CFI	TLI	RMSEA	SRMR
One-factor model	All items loaded on a single factor	1684.572	405	4.159	0.621	0.587	0.098	0.093
Two-factor model	AAF, PL, and ADQ combined; AISE and CO combined	1242.318	403	3.082	0.754	0.728	0.079	0.078
Three-factor model	AAF and PL combined; ADQ separate; AISE and CO combined	948.441	400	2.371	0.856	0.839	0.064	0.061
Four-factor model	AAF and PL combined; ADQ, AISE, and CO separate	732.905	398	1.842	0.921	0.910	0.052	0.046
Five-factor model (hypothesized)	AAF, PL, ADQ, AISE, and CO modeled as distinct factors	612.384	395	1.550	0.962	0.956	0.041	0.036

As an additional replicability check, measurement invariance was examined across academic level (undergraduate vs. postgraduate) and AI-library usage frequency groups. Configural and metric invariance were supported, as changes in CFI were below 0.010 across comparisons, indicating that the key constructs were measured consistently across these groups.

The structural model results are presented in Table 7. Consistent with H1, algorithmic academic framing had a significant positive effect on personalized learning (*β* = 0.584, SE = 0.051, CR = 11.451, *p* = 0.000, 95% CI [0.484, 0.684]). The confidence interval did not include zero, indicating a robust first-stage effect. Thus, H1 was supported. The structural model explained 54.8% of the variance in personalized learning (R^2^ = 0.548).

**Table 7:** Structural model results.

Path	β	SE	CR	*p*	95% CI (LL, UL)	f^2^	R^2^
Direct effects							
AAF → PL	0.584	0.051	11.451	0.000	0.484, 0.684	0.413	0.548
PL → ADQ	0.462	0.059	7.831	0.000	0.346, 0.578	0.238	0.612
AAF → ADQ	0.219	0.062	3.532	0.000	0.097, 0.341	0.064	
AISE → PL	0.271	0.048	5.646	0.000	0.177, 0.365	0.102	
CO → ADQ	−0.248	0.057	−4.351	0.000	−0.360, −0.136	0.081	
Moderation effects							
AAF × AISE → PL	0.143	0.039	3.667	0.000	0.066, 0.220	0.028	
PL × CO → ADQ	−0.128	0.041	−3.122	0.002	−0.208, −0.048	0.021	
Indirect, conditional indirect, and index of moderated mediation effects (bootstrapped, 5,000 resamples)							
AAF → PL → ADQ (indirect)	0.270	0.044	—	0.000	0.186, 0.356		
Indirect effect (high AISE, low CO)	0.341	0.051	—	0.000	0.242, 0.441		
Indirect effect (low AISE, high CO)	0.169	0.039	—	0.000	0.093, 0.245		
Index of moderated mediation (AISE)	0.066	0.020	—	0.001	0.027, 0.105		
Index of moderated mediation (CO)	−0.075	0.025	—	0.003	−0.124, −0.026		

*N* = 328. Bootstrap samples = 5,000. β = standardized coefficient. CI = confidence interval.

In line with H2, personalized learning significantly predicted academic decision-making quality (*β* = 0.462, SE = 0.059, CR = 7.831, p = 0.000, 95% CI [0.346, 0.578]). The positive coefficient and confidence interval excluding zero provide strong evidence that higher personalized learning is associated with improved academic decision quality. Therefore, H2 was supported. Collectively, the structural model explained 61.2% of the variance in academic decision-making quality (R^2^ = 0.612).

Regarding H3, algorithmic academic framing retained a significant direct effect on academic decision-making quality (*β* = 0.219, SE = 0.062, CR = 3.532, *p* = 0.000, 95% CI [0.097, 0.341]), suggesting partial mediation. The indirect effect through personalized learning was also significant (*β* = 0.270, 95% CI [0.186, 0.356], *p* = 0.000). Because both direct and indirect paths remained significant, personalized learning partially mediated the relationship, supporting H3.

For the moderation hypotheses, H4 predicted that AI self-efficacy would strengthen the relationship between algorithmic academic framing and personalized learning. The interaction term was positive and significant (*β* = 0.143, SE = 0.039, CR = 3.667, *p* = 0.000, 95% CI [0.066, 0.220]). The confidence interval excluded zero, confirming that the effect of algorithmic academic framing on personalized learning becomes stronger at higher levels of AI self-efficacy. Thus, H4 was supported.

H6 proposed that cognitive overload would weaken the relationship between personalized learning and academic decision-making quality. The interaction effect was negative and significant (β = −0.128, SE = 0.041, CR = −3.122, *p* = 0.002, 95% CI [−0.208, −0.048]), indicating that high cognitive overload attenuates the positive effect of personalized learning on decision quality. Therefore, H6 was supported.

Additional moderation diagnostics showed that the algorithmic academic framing × AI self-efficacy interaction increased the explained variance in personalized learning from R^2^ = 0.529 to R^2^ = 0.548 (ΔR^2^ = 0.019). Similarly, the personalized learning × cognitive overload interaction increased the explained variance in academic decision-making quality from R^2^ = 0.596 to R^2^ = 0.612 (ΔR^2^ = 0.016). These incremental changes indicate that both interaction terms added explanatory value beyond the main-effect models.

Finally, conditional indirect effect analysis showed that the indirect effect of algorithmic academic framing on academic decision-making quality via personalized learning was strongest when AI self-efficacy was high and cognitive overload was low (*β* = 0.341, 95% CI [0.242, 0.441], *p* = 0.000) (supporting H5), and weakest when AI self-efficacy was low and cognitive overload was high (*β* = 0.169, 95% CI [0.093, 0.245], *p* = 0.000) (supporting H7).

To evaluate moderated mediation, the index of moderated mediation was also estimated using 5,000 bootstrap resamples. The index for AI self-efficacy was positive and significant (*β* = 0.066, SE = 0.020, 95% CI [0.027, 0.105]), indicating that AI self-efficacy significantly strengthened the indirect effect of algorithmic academic framing on academic decision-making quality through personalized learning. The index for cognitive overload was negative and significant (*β* = −0.075, SE = 0.025, 95% CI [−0.124, −0.026]), indicating that cognitive overload significantly weakened this indirect effect. Because neither bootstrap confidence interval included zero, the moderated mediation results further supported H5 and H7. Collectively, the findings support the proposed conditional process model.

**Figure 2:**
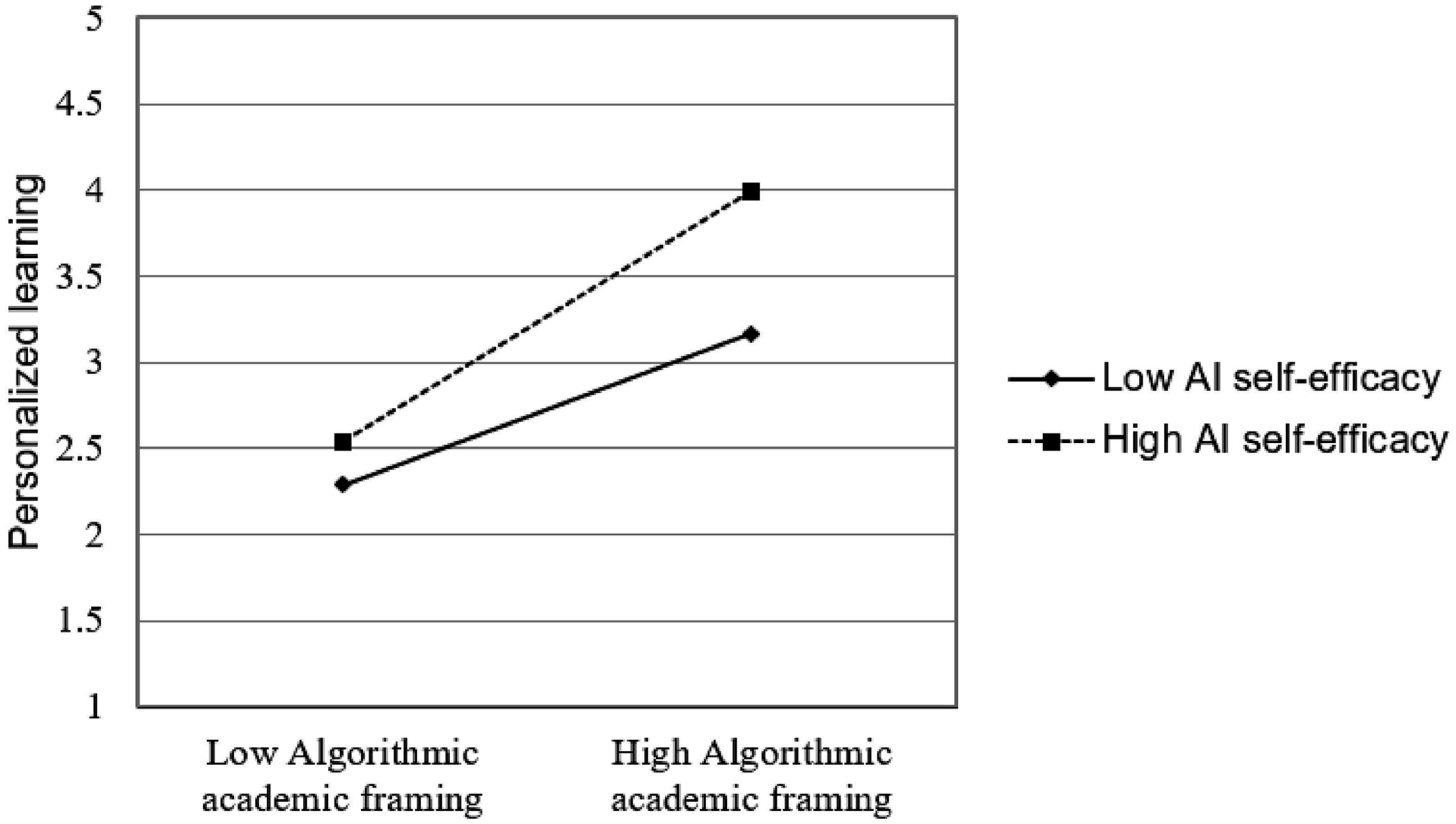
Interaction effect of AI self-efficacy x algorithmic academic framing on personalized learning.

**Figure 3:**
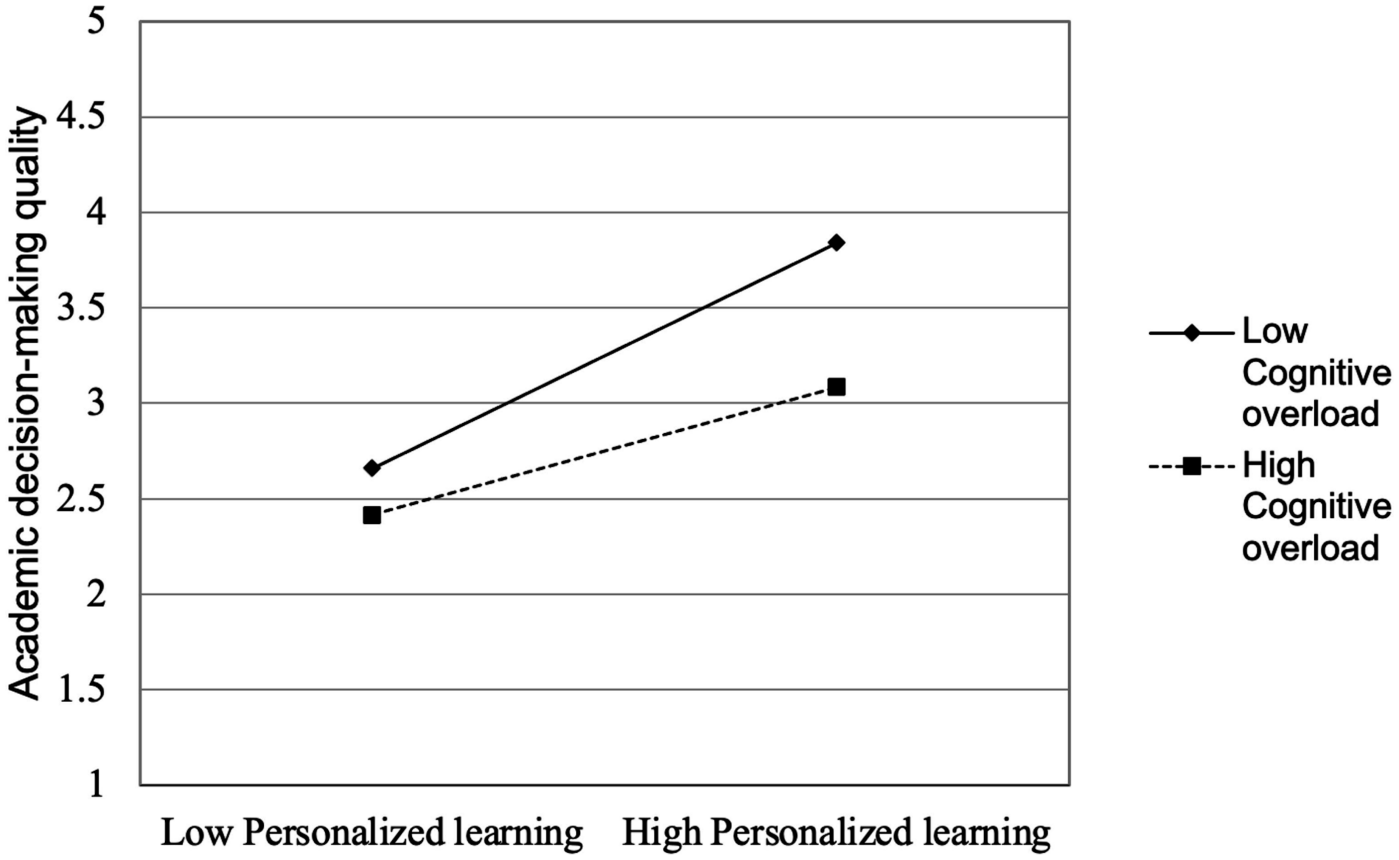
Interaction effect of cognitive overload x personalized learning on academic decision-making quality.

Figures 2, 3 depict the significant interaction effects identified in the structural model. As shown in Figure 2, the simple slope analysis revealed that the positive relationship between algorithmic academic framing and personalized learning was significantly stronger when AI self-efficacy was high compared to when AI self-efficacy was low. Following the conventional approach, simple slopes were estimated at one standard deviation above and below the mean level of the moderator. The effect of algorithmic academic framing on personalized learning was stronger under high AI self-efficacy conditions and weaker under low AI self-efficacy conditions, confirming the strengthening role of AI self-efficacy. This pattern indicates that students who are more confident in using AI-driven systems are better able to leverage algorithmic framing into meaningful personalized learning experiences.

In contrast, Figure 3 demonstrates that cognitive overload significantly attenuates the positive effect of personalized learning on academic decision-making quality. When cognitive overload was low, personalized learning strongly predicted decision quality; however, this effect was substantially weaker under high cognitive overload. Simple slope analysis at one standard deviation above and below the mean of cognitive overload confirmed that the positive association between personalized learning and academic decision-making quality remained stronger under low-overload conditions and was reduced when cognitive overload was high. Given that the latent interaction analysis was conducted within AMOS using the product-indicator approach, moderation was evaluated through latent interaction coefficients, conditional effects, and conventional simple slope probing.

To assess the robustness of the proposed model, additional analyses were conducted by including gender, academic level, academic discipline, and AI usage frequency as control variables. The inclusion of these controls did not materially alter the magnitude, direction, or statistical significance of the hypothesized relationships (see Appendix B, Panel A). Additional diagnostic checks also supported the robustness of the findings (see Appendix B, Panel B). The interaction terms added explanatory value, with ΔR^2^ = 0.019 for the AAF × AI self-efficacy interaction and ΔR^2^ = 0.016 for the personalized learning × cognitive overload interaction. Collinearity diagnostics indicated that VIF values ranged from 1.18 to 2.41 (see Appendix B, Panel C), and inspection of standardized residuals showed that most residuals were within ±2.58. Site-based robustness checks further indicated that the key paths remained directionally stable across the three universities. Therefore, the primary structural model is reported without control variables for the sake of parsimony.

## Discussion

The findings provide empirical support for the proposed conditional process model by demonstrating that algorithmic academic framing is associated with academic decision-making through personalized learning, while this process is simultaneously enabled and constrained by individual capability and cognitive resources. Specifically, the results indicate that AI self-efficacy strengthens the positive association between algorithmic academic framing and personalized learning, suggesting that students who feel competent in navigating AI-driven systems are better able to translate algorithmic cues into meaningful, tailored learning experiences. Conversely, cognitive overload weakens the positive association between personalized learning and academic decision-making quality, highlighting that excessive informational or mental burden may reduce students’ ability to effectively utilize personalized recommendations when making academic choices. Together, these findings suggest that AI-based smart libraries may function as empowering cognitive tools under conditions of high self-efficacy and manageable cognitive demand, whereas their potential benefits may be diminished when users experience cognitive strain. This nuanced understanding advances the literature by illustrating that the relationship between algorithmic systems and academic decision-making in higher education is not uniformly positive; rather, it appears to depend on users’ perceived capability and cognitive processing capacity.

The integration of cognitive load theory and social cognitive theory further suggests that AI-driven smart library systems should be understood not only in terms of technological functionality but also through the interaction between cognitive processing mechanisms and individual capability. Consistent with cognitive load theory, personalized learning may support more efficient processing of algorithmically organized information (Sweller, 2011), whereas social cognitive theory indicates that learners with stronger AI self-efficacy are better positioned to benefit from such environments (Bandura, 1986; Compeau and Higgins, 1995). Conversely, excessive cognitive demands may limit these potential benefits, reinforcing the complementary roles of AI self-efficacy and cognitive overload within the proposed framework.

### Theoretical implications

The current findings contribute to existing literature in several ways. For example, the empirical evidence that algorithmic academic framing is positively associated with personalized learning resonates with earlier scholarship demonstrating that algorithmic systems shape users’ evaluative judgments and information hierarchies (Domínguez Figaredo, 2020; Peng and Li, 2025). Prior research on recommendation engines and digital platforms has argued that algorithms subtly structure attention and define what appears salient or credible (Hardaker and Glenn, 2025). Our findings build on this foundation by situating algorithmic influence within academic environments and empirically demonstrating that such framing mechanisms are associated with enhanced personalization experiences. Unlike earlier studies that primarily emphasized user engagement or perceived usefulness (Chen and Perez, 2023; Sajja et al., 2025; Tariq, 2025), the present study conceptualizes AI-driven smart library systems as cognitive framing mechanisms that shape students’ interpretation of academic relevance and credibility rather than merely technological tools that deliver personalized recommendations. This perspective extends existing personalization and algorithmic influence research by emphasizing how AI systems may influence academic sensemaking in addition to facilitating information access.

Moreover, the mediating role of personalized learning offers an important theoretical bridge between AI system design and academic decision outcomes. While existing research has consistently shown that adaptive learning technologies improve perceived relevance and task efficiency (Tariq, 2025; Taylor et al., 2025), few studies have explicitly linked personalization to the quality of consequential academic decisions. For example, previous investigations reported improvements in engagement and satisfaction through personalization (Upadhyay, 2025; Venkateswaran, 2023), yet stopped short of examining downstream decision processes. By demonstrating that personalized learning partially explains how algorithmic framing improves decision-making quality, our findings extend personalization research into the domain of cognitive evaluation and strategic academic choice (Meng et al., 2025; Sajja et al., 2025). Moreover, the finding of partial mediation suggests that personalized learning represents one, but not the only, pathway linking algorithmic academic framing with academic decision-making quality. The remaining direct association indicates that additional mechanisms—such as perceived information credibility, trust in AI-generated recommendations, reduced search effort, or increased confidence in academic judgments—may also contribute to students’ academic decision processes. Future research should examine these complementary pathways to develop a more comprehensive understanding of algorithmic influence in higher education. More specifically, the direct path suggests that algorithmic academic framing may influence academic decision-making quality not only by increasing personalized learning, but also by shaping students’ perceived credibility of recommended sources, trust in AI-generated rankings, confidence in academic judgments, and perceived reduction of search costs. For example, when smart library systems present certain sources as more relevant or credible, students may experience greater decision confidence even before personalized learning is fully developed. Similarly, algorithmic transparency may help students understand why particular resources are recommended, thereby strengthening their confidence in selecting academic materials. These possibilities indicate that future research could test trust in recommendations, perceived credibility, decision confidence, reduced search effort, and algorithmic transparency as additional mediating mechanisms linking algorithmic academic framing with academic decision-making quality.

Recent research on ChatGPT and academic integrity in Hong Kong higher education further suggests that academic decision quality in AI-mediated environments may also depend on integrity-relevant norms and institutional policy signals, including policy awareness, perceived detection effectiveness, student integrity, and prior academic performance (Lo and Chan, 2025a). Similarly, research on generative AI in academic writing assessment shows that AI feedback and assistive features can shape learners’ writing strategies and evaluative standards in concrete assessment contexts (Lo and Chan, 2025b). These studies suggest that algorithmic academic framing operates not only through cognitive and personalization mechanisms, but also within broader institutional and assessment ecosystems.

The strengthening effect of AI self-efficacy further echoes established findings within social cognitive research showing that individuals’ confidence in technology use predicts enhanced system performance outcomes (Bergdahl and Sjöberg, 2025). Earlier studies observed that users with higher self-efficacy exhibit greater persistence, deeper engagement, and stronger learning gains when interacting with digital tools (Wang et al., 2023; Wang and Chuang, 2024). Consistent with this stream, our results indicate that AI self-efficacy amplifies the positive influence of algorithmic academic framing on personalized learning. However, the current study extends prior work by positioning AI self-efficacy not merely as an antecedent of technology adoption but as a boundary condition that strengthens the positive association between algorithmic academic framing and personalized learning (Rodríguez-Ruiz et al., 2025). This reconceptualization adds nuance to existing capability-based explanations of technology effectiveness.

At the same time, the moderating role of cognitive overload reinforces long-standing arguments that excessive informational demands undermine learning and judgment quality (Hong and Guo, 2025; Twabu, 2025). Research in multimedia learning and digital environments has repeatedly cautioned that information saturation can diminish comprehension and impair decision processes (Zha et al., 2023). Our findings corroborate these insights while extending them into AI-driven library contexts. Specifically, we demonstrate that cognitive overload weakens the positive association between personalized learning and academic decision-making quality, suggesting that personalization benefits are contingent upon manageable cognitive demand. By integrating empowerment (self-efficacy) and constraint (overload) perspectives within a single conditional framework, the study advances the literature beyond unidirectional models and highlights the complex, context-sensitive nature of algorithmic influence in higher education.

At the same time, the findings should not be interpreted as suggesting that algorithmic academic framing is uniformly beneficial. Although AI-driven smart libraries may support personalized learning and academic decision-making, excessive reliance on algorithmically generated recommendations could reduce students’ exposure to diverse academic perspectives, reinforce existing information preferences, or increase susceptibility to algorithmic bias. Consequently, AI-enabled educational systems should balance personalized guidance with opportunities for independent information exploration and critical evaluation, thereby supporting both learning efficiency and intellectual autonomy.

### Practical implications

The findings offer several important practical implications for university administrators, library managers, and educational technology designers. First, the positive impact of algorithmic academic framing on personalized learning and decision quality suggests that AI-driven smart libraries should be intentionally designed to provide transparent, explainable, and pedagogically aligned recommendation systems. Rather than simply optimizing search speed or volume of results, developers should focus on how algorithmic cues structure students’ academic attention and prioritization. For example, AI-enabled recommendation systems that suggest relevant journal articles based on users’ research interests, adaptive search functions that refine search results according to previous queries and academic objectives, and automated summarization tools that generate concise overviews of lengthy scholarly articles can help students navigate complex information environments more efficiently. Providing contextual explanations for why certain sources are recommended and allowing users to adjust personalization parameters may strengthen the constructive framing effect identified in this study. In addition, institutions should consider embedding AI literacy and training workshops within library orientations to enhance students’ AI self-efficacy and broader digital literacy, thereby maximizing the cognitive benefits of smart library systems (Jiang et al., 2023; Wang and Chuang, 2024; Wang et al., 2023).

Moreover, smart library platforms should balance personalization with exploration. While personalized recommendations can improve relevance and efficiency, systems should also expose students to diverse sources, alternative viewpoints, and adjacent research areas to reduce filter-bubble risks. Designers can support this balance by adding “explore beyond your topic,” “alternative perspectives,” or “why this was recommended” features. Recommendation interfaces should also provide explainability and controllability, allowing students to adjust recommendation criteria, change filters, and understand why specific sources are prioritized.

At the same time, the constraining role of cognitive overload underscores the need for careful interface design and workload management. Library platforms should avoid excessive recommendation density, redundant alerts, or overly complex dashboards that may overwhelm users. Features such as simplified search modes, progressive disclosure of information, AI-generated article summaries, and customizable filtering options can help reduce cognitive strain while preserving personalization benefits. Furthermore, instructors and academic advisors should guide students in strategically using AI tools rather than relying on them passively, ensuring that algorithmic assistance supports rather than substitutes critical evaluation. By balancing personalization with cognitive manageability, universities can harness AI-driven smart libraries as empowering academic infrastructures rather than sources of informational fatigue.

Universities may also establish structured human–AI workflows within library-supported research activities, in which AI systems assist with resource discovery, literature mapping, and preliminary organization, while librarians and instructors guide students in evaluating credibility, methodological relevance, and scholarly quality. Such collaboration can ensure that algorithmic support enhances, rather than replaces, information literacy skills and critical academic judgment.

Beyond technical and pedagogical considerations, the findings also highlight several ethical implications for the implementation of AI-driven smart libraries. Universities and technology developers should prioritize algorithmic transparency by providing users with clear explanations of how recommendations, rankings, and summaries are generated. Such transparency can help students critically evaluate AI-generated suggestions rather than accepting them uncritically. In addition, developers should regularly monitor AI systems for potential algorithmic bias to ensure equitable access to diverse academic perspectives and minimize unintended reinforcement of existing preferences.

Institutions should also align smart-library AI use with clear academic integrity and assessment policies. Policy awareness, disclosure expectations, and perceptions of detection effectiveness can shape whether students use AI support responsibly or problematically (Lo and Chan, 2025a). Therefore, library orientations and AI-literacy training should include guidance on acceptable AI use, source evaluation, disclosure norms, and assessment expectations, especially in writing-intensive academic tasks where generative AI feedback may influence students’ evaluative standards and learning strategies (Lo and Chan, 2025b).

Finally, AI-driven smart libraries should be designed to support, rather than replace, students’ independent judgment and critical thinking, as broader AI decision-support research suggests that AI-guided systems can shape users’ psychological and evaluative outcomes in sensitive decision contexts (Wang et al., 2026).

### Limitations

Despite its contributions, the study has several limitations that open avenues for future research. First, the study employed a purposive sampling strategy by recruiting students who had prior experience using AI-driven smart library systems. Although this approach was appropriate for examining algorithmic academic framing, it may have resulted in the overrepresentation of students who were already familiar with AI technologies and relatively comfortable interacting with such systems. Moreover, because the study was conducted in three Chinese universities where AI-driven smart library technologies are comparatively well established, the findings should be interpreted within this educational and technological context. Differences in AI adoption, digital infrastructure, institutional support, and students’ digital literacy across countries may influence the extent to which these findings generalize to other higher education settings. Consequently, the findings may not fully generalize to students with limited or no prior experience using AI-enabled academic platforms or to institutions with lower levels of AI integration. Future research should replicate the proposed model using more heterogeneous samples across different educational contexts and varying levels of AI adoption to further establish the generalizability of the findings. Second, the use of self-reported measures raises the possibility of perceptual bias, even though the time-lagged design helps mitigate common method concerns; subsequent research could incorporate objective behavioral data, such as system usage logs or academic performance indicators, to strengthen causal inference. Third, cognitive overload was operationalized as students’ general perceived overload when interacting with AI-driven smart library systems. Although this approach was appropriate for capturing overall cognitive strain arising from excessive information, recommendation density, and interface complexity, it did not separately distinguish intrinsic, extraneous, and germane cognitive load. Therefore, the findings should be interpreted as reflecting perceived overall overload rather than specific cognitive load components. Future research should replicate the model using multidimensional cognitive load instruments, such as Leppink et al.’s cognitive load scale or adapted NASA-TLX measures, to examine whether different forms of cognitive load differentially condition the effect of personalized learning on academic decision-making quality. Fourth, the model focused on supportive forms of algorithmic academic framing, whereas future investigations might explore potentially negative framing effects, such as algorithmic bias or over-reliance, to provide a more balanced understanding of AI influence. Finally, longitudinal or experimental designs could further clarify causal mechanisms and examine how the empowerment–constraint dynamics evolve over time as students gain experience with AI-driven smart library systems.

## Conclusion

In conclusion, this study advances understanding of how AI-driven smart libraries are associated with academic outcomes by introducing algorithmic academic framing as a central explanatory mechanism. The findings suggest that algorithmic academic framing is positively associated with academic decision-making quality indirectly through personalized learning, while the strength of this association depends on students’ AI self-efficacy and cognitive overload. By integrating empowerment and constraint perspectives within a single conditional framework, the study moves beyond simplistic assumptions of uniformly positive AI effects and highlights the nuanced, context-dependent nature of algorithmic influence in higher education. Overall, the results emphasize that the potential effectiveness of AI-driven academic infrastructures appears to depend not only on technological sophistication but also on users’ capability and cognitive capacity, offering a more comprehensive account of how AI-driven smart libraries may be associated with contemporary academic decision processes. Given the observational nature of the study, these findings should be interpreted as associative rather than causal, and future longitudinal and experimental research is needed to establish causal relationships more rigorously.

## Data Availability

The raw data supporting the conclusions of this article will be made available by the authors, without undue reservation.
