# Supplementary material for: Algorithmic academic framing in AI-driven smart libraries: a moderated mediation model of personalized learning and cognitive overload in academic decision-making
Source: Front Psychol. 2026 Jul 31;17:1864163. doi: 10.3389/fpsyg.2026.1864163 (PMC13475612; doi:10.3389/fpsyg.2026.1864163)
Supplement: Supplementary file 1 [file Supplementary_file_1.docx]

**Appendix A. Measurement Items**

**Algorithmic Academic Framing**

**AAF1.** The AI-driven smart library influences how I prioritize academic sources.

**AAF2.** Recommendations provided by the AI-driven smart library shape which academic materials I consider most relevant.

**AAF3.** AI-generated rankings influence my evaluation of the credibility of academic resources.

**AAF4.** Suggestions from the AI-driven smart library guide the topics or research directions I choose to explore.

**AAF5.** AI-generated summaries influence how I interpret the importance of academic information.

**AAF6.** The AI-driven smart library shapes my academic decision-making by emphasizing certain information over others.

**Personalized Learning**

**PL1.** The AI library system provides learning resources that match my academic interests.

**PL2.** The AI library system recommends materials that fit my learning needs.

**PL3.** The AI library system helps me locate resources relevant to my academic goals.

**PL4.** The recommendations provided by the AI library system support my individual learning process.

**PL5.** Overall, the AI library system provides a personalized learning experience.

**Academic Decision-Making Quality**

**ADQ1.** I make well-informed academic decisions when selecting research materials.

**ADQ2.** I carefully evaluate academic information before making study-related decisions.

**ADQ3.** I am confident in the quality of my academic decisions.

**ADQ4.** I usually select academic resources that best support my learning objectives.

**ADQ5.** Overall, I make effective academic decisions when using AI-driven library systems.

**AI Self-Efficacy**

**AISE1.** I feel confident in my ability to use AI-based library tools effectively.

**AISE2.** I can successfully use AI-driven library functions even without assistance.

**AISE3.** I am capable of learning new AI-based library features when needed.

**AISE4.** I believe I can effectively use AI-driven smart library systems for academic tasks.

**Cognitive Overload**

**CO1.** Using the AI library system sometimes overwhelms me with too much information.

**CO2.** I often receive more information from the AI library system than I can effectively process.

**CO3.** I find it mentally demanding to evaluate all the recommendations provided by the AI library system.

**CO4.** The amount of information presented by the AI library system sometimes makes academic decision-making difficult.

**Appendix B.** Robustness analyses and diagnostic checks

**Panel A.** Robustness analysis including control variables

| **Control variable** | **Outcome** | **β** | **p** |
| --- | --- | --- | --- |
| Gender | PL | .031 | .472 |
| Academic level | PL | .047 | .281 |
| Discipline | PL | -.026 | .536 |
| AI usage frequency | PL | .082 | .071 |
| Gender | ADQ | .024 | .558 |
| Academic level | ADQ | .038 | .331 |
| Discipline | ADQ | -.035 | .389 |
| AI usage frequency | ADQ | .091 | .054 |

**Panel B.** Additional diagnostic checks

| **Diagnostic check** | **Result** | **Interpretation** |
| --- | --- | --- |
| AAF × AI self-efficacy ΔR² | .019 | Interaction adds explanatory value for PL |
| PL × cognitive overload ΔR² | .016 | Interaction adds explanatory value for ADQ |
| Missing-data robustness | Little’s MCAR test was non-significant, χ² = 41.27, df = 38, p = .326; FIML-based estimates were substantively consistent with listwise-deletion results | Findings were not sensitive to missing-data handling |
| Measurement invariance | Configural and metric invariance supported across academic level and AI-library usage frequency groups; ΔCFI < .010 | Key constructs showed acceptable stability across groups |
| Standardized residuals | Most residuals within ±2.58 | No substantial residual misspecification |
| University-site robustness | Key paths remained directionally stable across the three universities | Findings were not driven by one institutional subgroup |

**Panel C.** Collinearity diagnostics

| **Predictor** | **VIF** |
| --- | --- |
| Algorithmic academic framing | 1.74 |
| Personalized learning | 2.41 |
| AI self-efficacy | 1.63 |
| Cognitive overload | 1.38 |
| AAF × AI self-efficacy | 2.12 |
| PL × cognitive overload | 2.26 |
| Gender | 1.18 |
| Academic level | 1.31 |
| Discipline | 1.22 |
| AI usage frequency | 1.46 |
